# Telomere Length, Proviral Load and Neurologic Impairment in HTLV-1 and HTLV-2-Infected Subjects

**DOI:** 10.3390/v8080221

**Published:** 2016-08-11

**Authors:** Benjamin Usadi, Roberta Bruhn, Jue Lin, Tzong-Hae Lee, Elizabeth Blackburn, Edward L. Murphy

**Affiliations:** 1 School of Public Health, University of California Berkeley, Berkeley, CA 94720-7360, USA; benusadi@gmail.com; 2 Blood Systems Research Institute, San Francisco, CA 94118, USA; rbruhn@bloodsystems.org (R.B.); tlee@bloodsystems.org (T.-H.L.); 3 Departments of Biochemistry and Biophysics, University of California San Francisco, San Francisco, CA 94158, USA; Jue.Lin@ucsf.edu (J.L.); eblackburn@salk.edu (E.B.); 4 Salk Institute for Biological Studies, La Jolla, CA 92037, USA; 5 Laboratory Medicine, University of California San Francisco, San Francisco, CA 94158, USA; 6 Epidemiology and Biostatistics, University of California San Francisco, San Francisco, CA 94158, USA

**Keywords:** HTLV, HAM, telomere

## Abstract

Short or damaged telomeres have been implicated in degenerative conditions. We hypothesized that analysis of telomere length (TL) in human T-cell lymphotropic virus (HTLV) infection and HTLV-associated neuropathy might provide clues to the etiology of HTLV-associated disease and viral dynamics. A subset of 45 human T-cell lymphotropic virus type 1 (HTLV-1), 45 human T-cell lymphotropic virus type 2 (HTLV-2), and 45 seronegative subjects was selected from the larger HTLV Outcomes Study (HOST) cohort, matched on age, sex and race/ethnicity. Telomere-to-single-copy gene (T/S) ratio (a measure of TL) and HTLV-1 and HTLV-2 proviral loads were measured in peripheral blood mononuclear cells (PBMCs) using quantitative PCR (qPCR). Vibration sensation measured by tuning fork during neurologic examinations performed as part of the HOST study allowed for an assessment of peripheral neuropathy. TL was compared between groups using *t*-tests, linear and logistic regression. Mean T/S ratio was 1.02 ± 0.16 in HTLV-1, 1.03 ± 0.17 in HTLV-2 and 0.99 ± 0.18 in HTLV seronegative subjects (*p* = 0.322). TL was not associated with HTLV-1 or -2 proviral load. Shorter TL was significantly associated with impaired vibration sense in the HTLV-2 positive group only. Overall, we found no evidence that telomere length was affected by chronic HTLV-1 and HTLV-2 infection. That TL was only associated with peripheral neuropathy in the HTLV-2-positive group is intriguing, but should be interpreted cautiously. Studies with larger sample size and telomere length measurement in lymphocyte subsets may clarify the relationship between TL and HTLV-infection.

## 1. Introduction

Telomeres, the repetitive nucleoprotein complexes that protect chromosome ends from damage, can shorten with each cell replication. If telomere length decreases beyond a critical threshold, normal cells typically enter a senescent state or can be directed to an apoptotic pathway. Hence, telomeres have been termed “clocks” that count down with each cell cycle, marking progress through the replicative lifespan of individual cells, unless they are rebuilt, usually by the telomere DNA synthesizing enzyme telomerase.

Human T-cell lymphotropic virus type 1 (HTLV-1) and type 2 (HTLV-2) infection has been associated with increased all-cause mortality [1,2,3,4] and infections [5], as well as degenerative neurologic disease such as HTLV-associated Myelopathy (HAM) and more subtle neurologic impairment [6]. A body of evidence suggests a role for oxidative stress in a variety of degenerative diseases [7,8,9]. HTLV-associated myelopathy, also known as tropical spastic paraparesis (HAM/TSP), is an inflammatory condition that has been associated with a pattern of enhanced expression of certain interferon-stimulated genes [10]. Previous research indicates that short telomere length (TL) is associated with degenerative age-related diseases [11], such as osteoarthritis [12] and cardiovascular disease [13,14,15]. Shorter telomere length in individuals has also been associated with higher rates of mortality [16]. As cells divide throughout the aging process, telomeres shorten with each DNA replication and continued shortening may induce cellular senescence or apoptosis.

Because HTLV proviral DNA integrates into the host chromosome and promotes cell division as part of a replication strategy, TL in infected cells could be affected as a result: either rapidly shortening TL or altering TL regulation to promote survival of HTLV-infected cells, similar to cancer cells where reactivation of telomerase maintains cells with shorter telomeres, thus contributing to genomic instability and cellular malfunction) [16,17]. HTLV tax gene expression has also been shown to be intimately connected with telomerase, giving further credence to the hypothesis that HTLV-infection may alter telomere length [18]. Bellon, et al. describe a non-monotonic relationship between HTLV infection and telomerase gene regulation that they postulate may facilitate persistence of altered T-cells [19]. Zane, et al. found evidence of altered telomerase activity associated with HTLV infection (using samples derived from uninfected controls and HTLV-infected subjects with either HAM/TSP or adult T-cell leukemia/lymphoma (ATLL)) [20], although Sinha-Datta, et al. did not find evidence of altered human telomerase reverse transcriptase expression in cells derived from asymptomatic HTLV-1 carriers [21]. Although HTLV is not known to directly infect neuronal cells, altered telomerase activity and perpetuation of T-cells could contribute to neurological degeneration by facilitating an inflammatory environment with concomitant oxidative stress.

Given the associations between TL, interferon-related gene expression [22], oxidative stress, and degenerative disease, together with the evidence of altered telomerase activity in HTLV-infected cells [20], we hypothesized that TL is associated with HTLV infection and HTLV-related disease symptoms. We performed a retrospective analysis of telomere length in a cohort of HTLV-1-positive, HTLV-2-positive, and HTLV-seronegative subjects who had been assessed for HTLV-associated neurologic impairment.

## 2. Materials and Methods

### 2.1. Study Design and Subjects

From the HTLV outcomes study (HOST, a United States-based cohort consisting of 151 HTLV-1, 387 HTLV-2 and 799 seronegative subjects enrolled after blood donation from 1990 to 1999 and followed for almost 20 years) [5], a subset of 45 HTLV-1, 45 HTLV-2, and 45 seronegative subjects from the baseline visit in 1990–1992 was selected, with group matching on age, sex and self-reported race/ethnicity. The University of California San Francisco Committee on Human Research approved this study (#10-00852). Detailed interviews of the study subjects were conducted, including background on demographic characteristics, smoking and alcohol consumption. In addition, standard laboratory analyses (differential CBC and lipid panel) and neurologic exams were performed. Nine study participants (one seronegative, three HTLV-1-positives and five HTLV-2-positives) had moved out of the area since enrollment and were not available for the neurologic examinations, although they participated in interviews and other laboratory measures. The neurologic examiners were not blinded to the subjects’ HTLV status, but followed standardized methods to record findings of tuning fork vibration sensation, a validated method for peripheral neuropathy screening [23,24]. These examiners classified subjects into three vibration sense categories: no vibration sensation; impaired vibration sensation (felt vibration four or more seconds shorter than the examiner); and no impairment (felt vibration less than four seconds shorter than the examiner).

### 2.2. Laboratory Measures

Fresh whole blood samples from HOST subjects, collected at study visits, were centrifuged to separate peripheral blood mononuclear cells (PBMC’s), which were washed and stored in 5 × 10^6^ cell aliquots in liquid nitrogen. Each PBMC aliquot that was analyzed for the current study was selected from the final HOST study visit, thawed and split, with one-half used for proviral load measurement and one-half used for telomere-to-single-copy gene (T/S) ratio (a relative measure of TL) measurement.

Telomere measurement was by quantitative PCR (qPCR) [25] at the Blackburn laboratory at the University of California San Francisco (UCSF), using a previously published method [26]. Genomic DNA was prepared from PBMCs using QIAamp DNA blood mini kit (cat#51106) (QIAGEN, Hilden, Germany). The T/S ratio was obtained by comparing the abundance of telomeric repeats to the abundance of a single copy gene (human β-globin) using genomic DNA from human cancer cell line HeLa as the reference standard. The quantity of targeted templates in each research sample was determined relative to the reference DNA sample by the standard curve method. The same reference DNA was used for all PCR runs. All samples were run in triplicate wells and the average values for the triplicates were used for calculating T/S ratio. To control for inter-assay variability, eight control DNA samples were included in each run. In each batch, the T/S ratio of each control DNA was divided by the average T/S for the same DNA from 10 runs to get a normalizing factor. This was done for all samples and the average normalizing factor for all samples was used to correct the participant DNA samples to get the final T/S ratio. Two initial measurements were performed on each sample, with an additional measurement performed if the first measurements diverged beyond 7%. In these cases, the average of the two most similar measurements was used as the final T/S measurement.

Proviral loads were measured using real-time qPCR amplification with primers common to both HTLV-1 and HTLV-2 and detection by SYBR Green (Thermo Fisher Scientific, Waltham, MA, USA), which allowed direct comparison of proviral loads between types 1 and 2. Type-specific TaqMan probes (Thermo Fisher Scientific, Waltham, MA, USA) were used to distinguish HTLV-1 from HTLV-2 provirus. The specific method has been previously published [27].

### 2.3. Statistical Analysis

Univariate analysis of subjects’ demographic characteristics, smoking and alcohol-use history, and laboratory measurements was performed to describe the study population groups. Proviral load was log_10_ transformed. Seropositive cases with no detectable virus were imputed with a proviral load of 1 per 10^6^ cells (which is the lower limit of detection) in order to allow for log transformation). Unpaired *t*-tests (with adjustment for unequal variances, as necessary) were performed to compare HTLV-1 and HTLV-2 cases and controls in terms of demographic characteristics, smoking and alcohol history, and telomere length (mean T/S ratio).

Unadjusted linear regression was used for an initial assessment of the dependence of telomere length (log_e_ T/S ratio) on proviral load (log_10_ transformed). Potential confounders, including age, sex, body mass index (BMI), smoking (cumulative pack-years), and alcohol use (drinks/week in previous two years) were evaluated for possible inclusion in an adjusted model, using a *p*-value < 0.10 as a threshold for consideration.

A binary vibration-sense outcome variable was created, with subjects classified as either impaired or not impaired with respect to their ability to detect tuning fork vibrations. Bivariate logistic regression was performed to assess for any association between impaired vibration sensation and log_e_-transformed T/S ratio, and other covariates.

Multivariable linear regression was used to assess the independent association of telomere length on proviral load (log_10_). Potential confounders were included in the final model based on biological plausibility and association with telomere length in bivariate analysis at significance of *p* < 0.10. Multivariable logistic regression was also performed to assess whether PBMC telomere length was predictive of vibration-sense impairment. Potential confounders identified in bivariate analysis (*p* < 0.10) were included in the adjusted model based on likelihood ratio tests and biological plausibility. Statistical analysis was performed using Stata (StataCorp, College Station, TX, USA, version 12.1).

## 3. Results

### 3.1. Study Population

Table 1 summarizes demographic and other characteristics of the three HTLV-status groups. Given group matching, demographic characteristics were generally similar between HTLV cases and controls. The seronegative group had lower cumulative lifetime smoking than either HTLV-positive group. Both the HTLV-1 and -2 positive groups had fewer members with income of $30,000 per year or more than did the control group, and also had fewer members with at least some college education when compared to controls. Eight subjects in the HTLV-2 group self-reported a history of injection drug use (IDU). Two HTLV-2-positives had been diagnosed with HAM/TSP, but neither was a former IDU. The proportion of subjects with impaired vibration sense did not differ significantly between seronegative, HTLV-1 and HTLV-2 groups (18%, 26% and 20%, respectively).

### 3.2. Telomere Length

**Figure 1:**
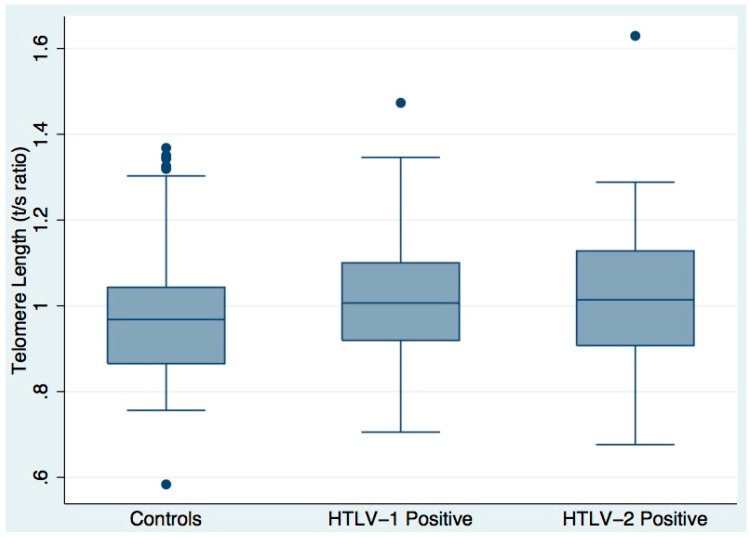
Telomere length by human T-cell lymphotropic virus (HTLV) infection status. Telomere-to-single-copy gene (T/S) ratio measured by quantitative PCR (qPCR) for 45 seronegative donor controls, 45 HTLV-1 positive patients and 45 HTLV-2 positive patients. Boxes show median and IQR, whiskers depict additional range, except for values > 1.5 × IQR from median, which are shown as circles. Mean T/S ratio (1.02 ± 0.16 in HTLV-1, 1.03 ± 0.17 in HTLV-2 and 0.99 ± 0.18 in HTLV seronegative subjects) did not differ significantly; comparing either HTLV-1 or HTLV-2 cases to controls, or combined HTLV cases to controls (*p* = 0.322).

No significant differences in mean telomere length were observed, comparing either HTLV-1 or HTLV-2 cases to controls, or combined HTLV cases to controls (Figure 1). Mean T/S ratio was 1.02 ± 0.16 in HTLV-1, 1.03 ± 0.17 in HTLV-2 and 0.99 ± 0.18 in HTLV seronegative subjects (*p* = 0.322, comparing combined cases to controls). In unadjusted regression analysis by HTLV-status (type 1, type 2, or seronegative), telomere length (loge T/S ratio) was significantly associated with age (inversely) only in the HTLV-negative control group (*p* = 0.007). Cumulative smoking (lifetime pack-years) was inversely associated with telomere length in the HTLV-1 cases (*p* = 0.009), but not associated within HTLV-2 cases or controls. Log_10_ proviral load had a borderline-significant association (*p* = 0.069) with telomere length only in the HTLV-1-positive group. Within the HTLV-2 cases, TL in subjects with HAM was shorter (mean T/S ratio 0.78 ± 0.15) than in those without (mean T/S ratio 1.04 ± 0.17), although the paucity of HAM cases precluded formal assessment of this difference.

In an analysis combining HTLV-1 and -2 cases and controls (Table 2), age (*p* = 0.003), smoking (*p* = 0.015) and sex (*p* = 0.097) reached the threshold (*p* < 0.10) for consideration as candidates in an adjusted regression model of log_e_ T/S ratio. Higher age and smoking and female sex were negatively associated with TL. In a multivariate model that adjusted for age, pack-years of smoking and sex, log_10_ proviral load was not associated with telomere length in either HTLV-1 or HTLV-2 cases. In the control group only, higher age remained significantly associated with lower log_e_ TL (*p* = 0.006) after adjustment for smoking and sex. In the HTLV-1 positive group, cumulative smoking history had a borderline significant inverse association with log_e_ T/S ratio (*p* = 0.052). There were no significant predictors of T/S ratio in adjusted HTLV-2-specific analysis.

### 3.3. Tuning Fork Sensation Impairment Associations

**Figure 2:**
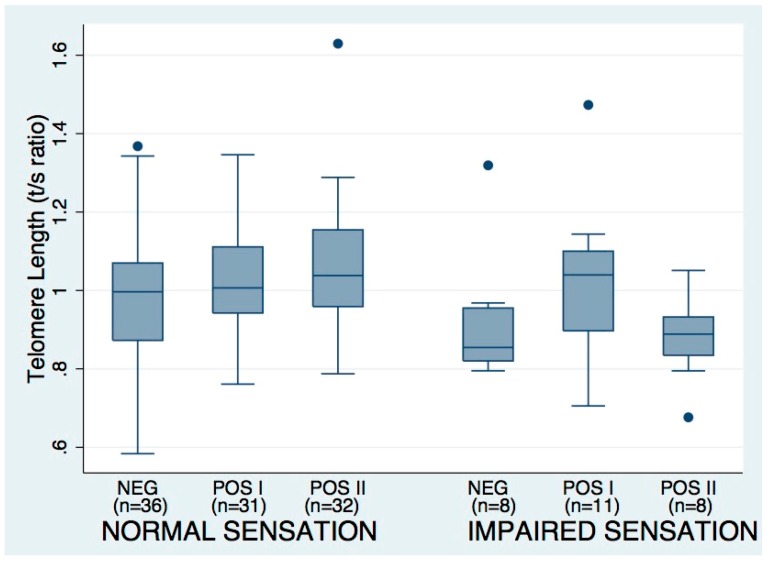
Telomere length by HTLV status and presence or absence of vibration sense impairment. T/S ratio measured by qPCR and vibration sensation measured by tuning fork. Boxes show median and IQR, whiskers depict additional range, except for values > 1.5 × IQR from median, which are shown as circles. NEG = seronegative controls; POS1 = HTLV-1 Positive; POS2 = HTLV-2 Positive.

Figure 2 conveys the distribution of telomere length by HTLV- and impaired sensation status. In bivariate analyses including all HTLV-1 and -2 cases and controls, the odds of impaired vibration sensation were increased with higher age (*p* = 0.025), greater cumulative smoking (*p* = 0.027) and lower log_e_ T/S ratio (*p* = 0.024; Table 3). Factors associated with impaired tuning fork sensation differed by HTLV-status. In the control group only, older age was associated with increased odds of tuning fork sensation impairment (*p* = 0.034). Shorter telomere length, the primary independent variable of interest with respect to neurologic outcomes, was associated with increased odds of tuning fork sensation impairment in the HTLV-2 cases (*p* = 0.014), but not in the HTLV-1 cases or controls. While the data are suggestive of seronegative subjects with impaired sensation demonstrating shorter TL when compared with seronegatives with normal sensation, the large variability results in lack of statistical difference.

In a logistic regression model that adjusted for age, smoking and log_e_ T/S ratio, the pattern of significant predictors of vibration sense impairment was markedly different among HTLV-1 and -2 cases and controls. In the control group, age did not retain its significant association with the odds of impaired vibration sensation after adjustment. Smaller log_e_ T/S ratio was significantly associated (*p* = 0.026) with increased odds of vibration-sensation impairment only in the HTLV-2-positive group.

## 4. Discussion

Our main hypothesis was not supported as telomere length did not differ by HTLV infection status and neither HTLV-1 or HTLV-2 proviral load was predictive of TL. However, we did observe that age effect was weaker in HTLV-1 and -2 cases compared to seronegative controls. Interestingly, shorter TL was associated with impaired vibration sense among HTLV-2 subjects only.

The failure to detect any significant differences in mean T/S ratio between cases and controls could have several explanations. First, our hypothesis that there is an underlying difference in the mean telomere lengths of the target groups may have been wrong. Telomere length is under several complex controls [14] and in HTLV infections, compensatory mechanisms for telomere length regulation, potentially (but not exclusively) including up-regulation of telomerase or epigenetic modification of the telomeres, could confer a selective advantage on cells in vivo. Age-related telomere shortening could be blocked by some viral effect on the multiple pathways of telomere regulation that makes them resistant to further attrition. Alternatively, if HTLV infection does lead to telomere shortening, the combination of age and HTLV effects may push the cells with shortest telomeres past the threshold for senescence/apoptosis, leading to the removal of the cells with shortest telomeres from the PBMC population prior to laboratory analysis. Second, the sample size of our study was relatively small, so an effect of very small magnitude may have been obscured. A previous study [17] found significantly shorter telomeres in HTLV-infected patients with ATL compared to both asymptomatic HTLV-positive subjects and seronegative controls. Similar to our findings, Kubuki, et al. did not find a significant difference in TL when comparing asymptomatic HTLV-infected subjects to negative controls although their study was also of limited size.

Consistent with previous studies and underlying theory age was inversely associated with telomere length in all subjects combined. But when subjects were separated by infection status, age retained this association only in the control group. Although most age-related telomere shortening occurs early in life [11], past research suggests a downward trend in telomere length as people age [28,29], even in the age range of these study participants. If no other factors were involved, the weakness of an age contribution to telomere shortening in the HTLV cases would be expected to manifest as differences in mean TL between HTLV cases and age-matched controls, which was not seen. The most likely explanation is smaller sample size on the subgroup analysis. However, it is conceivable that the lack of an age association in the HTLV subjects may be a weak signal of some of the biological mechanisms noted above.

That proviral load was not associated with telomere length in multivariable analysis may reflect the true lack of an association, compensatory mechanisms and in vivo cellular selection (as discussed previously), small sample size, a relatively low percentage of infected PMBCs in these subjects, or unaccounted confounding. There are several reasons to expect proviral load to be associated with telomere length in the PBMCs of HTLV-infected subjects. First, HTLV-provirus spread largely results from clonal expansion of infected cells, and telomeres shorten with each cell division. Therefore, it would be expected that higher proviral load would be associated with shorter telomeres. Recent research suggests that in HTLV-1-infected subjects, even the provirus-free T-cells have altered telomerase activity [20]. Hence, even individuals with low proviral load might have detectable differences in mean telomere length compared to uninfected subjects. Second, higher proviral loads have been associated with increased risk of HTLV-associated disease [30]. If telomere dysfunction is a mediator or biomarker of this relationship, then it would be expected that higher proviral load would be reflected in altered average telomere length. A similar lack of association of CMV viral load with TL has been reported [31] and in that study, CMV viral load was related to reduced basal telomerase activity in PBMCs, which was not measured in the current retrospective HTLV study.

It is interesting that only in the HTLV-1 group did proviral load and smoking show some evidence of association with TL in bivariate analysis, although only smoking remained as a borderline significant predictor (*p* = 0.052) after adjustment. Evidence from previous research suggests that smoking is indeed a risk factor for telomere attrition [16,32], but it is unclear why this association was not found in either the control or HTLV-2 positive groups. Research by Zane, et al. suggests that activated T-cells of HTLV-1 infected individuals have impaired telomerase activity [20]. This could prevent the rebuilding of telomeres that have been shortened by smoking-induced oxidative stress, resulting in a measurable association between smoking and TL in this group.

Given the inflammatory component of HTLV-associated neurologic disease, it is plausible that altered telomere length could serve as a biomarker of, or contributor to, immune processes that result in reactive oxygen species (ROS)-induced damage in impaired tuning fork sensation in this study population. Neurologic dysfunction due to HTLV infection has been associated with inflammation and altered cytokine expression [10,33]. There is evidence that other degenerative diseases, such as osteoarthritis and cardiovascular disease, have an immune-mediated inflammatory component with concomitant release of ROS [7,34]. Recent research also implicates ROS in peripheral neuropathy [35].

This study benefited from a well-characterized HTLV cohort with good collection of medical information. It utilized a telomere length assay performed in the laboratory of the discoverer of telomerase and a proviral load assay performed in the laboratory of the creator of the HTLV-specific qPCR protocol. While the number of male subjects is small (the epidemiology of HTLV is such that infection is more common in women and the HOST cohort is 70% female), the cases and controls were group-matched on age, sex, and self-reported race/ethnicity, and all subjects in this analysis were living in or near major population centers, which may have also helped to evenly distribute unknown confounders between the groups.

The major weaknesses of this study were the relatively small sample size, the lack of longitudinal telomere length measurements, and measurement of TL in unsorted PBMC rather than lymphocyte subsets. The sample size for the study was moderate and, in addition, a small number of subjects had moved out of the area since enrollment in the larger HOST cohort, further reducing the sample size for the peripheral neuropathy analysis. While the correlation between impaired tuning fork sensation and progression to HAM in HTLV-infected persons has not been demonstrated, Saeidi et al. reported that 30% of patients with HAM/TSP also experienced peripheral neuropathy [36]. Due to limited resources, we decided to measure TL at a single time-point in subjects who had been infected for unknown durations, and therefore could have missed a TL association that was present earlier in their HTLV infection. If a difference in TL had been observed, we intended to study prospectively acquired repository samples but our negative finding suggest this would be of low yield. Finally, we did not sort cells into CD4 and CD8 subsets; the PCR-based telomere measurements reflect average telomere length in total PBMCs. Only a small proportion of PBMCs carry provirus and clonal expansion of infected cells may have been insufficient to impact the T/S ratio of the total PBMC population; therefore, it is conceivable that we missed an association between HTLV status and TL that was limited to a subset of lymphocytes.

In conclusion, this study did not find any differences in mean TL between HTLV cases and controls but there was some evidence that the relationship between neuropathy and TL is different among HTLV-1-positive, HTLV-2 positive, and seronegative individuals. Since only a small proportion of HTLV-infected individuals progress to clinical disease, it would be interesting from an etiologic perspective, and possibly useful as a prognostic tool, to determine whether telomere length plays a role in the progression to neurologic disease. A study with larger sample size, CD4/CD8 subset sorting, longitudinal telomere length measurements, and measurement of additional potential confounders, such as diet and antioxidant intake, may better able to unravel any possible relationships between telomere length, HTLV-infection, and HTLV neurologic disease.

## Figures and Tables

**Table 1:** Characteristics of the study population.

	HTLV-NEG	HTLV-1 POS	HTLV-2 POS
	*n* = 45	*n* = 45	*n* = 45
**Age**			
35–45	3	3	3
45–55	12	12	12
>56	30	30	30
Mean, Median	58.5, 58.7	58.4, 59.1	60.1, 59.3
**Sex**			
Male	8	8	8
Female	37	37	37
**Race/Ethnicity**			
White	15	15	15
Black	22	22	22
Hispanic *	6	3	8
Japanese *	1	4	0
Native American *	1	1	0
**Country of Birth**			
USA	43	37	43
Caribbean	1	3	0
Central & South America	0	3	1
Mediterranean	1	1	0
Japan & Taiwan	0	2	1
**Geographic Region of Enrollment**			
Eastern US	10	11	10
Central US	16	16	8
Western US	19	18	27
**Smoking, Alcohol & Drug Use**			
% Current Smoker	6.7	22.2	17.8
% History of Smoking	46.7	43.2	68.9
Pack-years (mean, median)	5.3, 0	9.6, 0	9.1, 2
Drinks/week (mean, median)	2.0, 0.1	1.6, 0	1.3, 0.1
BMI (mean, median)	30.7, 29.7	30.2, 28.3	32.3, 31.6
History of Injection Drug Use	0	0	8
**Log_10_ Proviral Load**			
Median (Range)	NA	−3.79 (−6.00, −1.80)	−4.96 (−6.00, −2.33)
**Vibration Sensation**			
Impaired	8	11	8
Not Impaired	36	31	32
HAM/TSP	NA	0	2

* Grouped as “Other Race”; HTLV = human T-cell lymphotropic virus; NEG = negative for HTLV; POS = positive for either HTLV-1 or HTLV-2; HAM/TSP = HTLV-associated myelopathy/tropical spastic paraparesis.

**Table 2:** Variables associated with log_e_ (telomere-to-single-copy gene (T/S) ratio).

Group	Variable	Beta (Unadjusted)	*p*-Value	Beta (Adjusted)	*p*-Value *
**All subjects**	**Age**	−0.005	0.003	−0.004	0.007
	**Smoking**	−0.002	0.015	−0.001	0.076
	**Sex (male)**	0.061	0.097	0.050	0.165
**Controls**	**Age**	−0.008	0.007	−0.009	0.006
**HTLV-1**	**Log_10_ proviral load**	−0.032	0.069	−0.023	0.167
	**Smoking**	−0.003	0.009	−0.002	0.052
**HTLV-2**	**No significant associations**				

* Multivariable linear regression adjusted for age, smoking (pack-years), sex and log_10_ proviral load (where applicable).

**Table 3:** Variables associated with tuning fork sensation impairment.

Group	Variable	Odds Ratio	95% CI	Odds Ratio *	95% CI *
**All subjects**	**Age**	1.062	(1.008, 1.119)	1.046	(0.989, 1.106)
	**Smoking**	1.033	(1.004, 1.063)	1.026	(0.997, 1.057)
	**Log*_e_* T/S ratio**	0.714	(0.534, 0.956)	0.826	(0.610, 1.118)
**Controls**	**Age**	1.130	(1.009, 1.258)	1.129	(0.988, 1.292)
**HTLV-1**	**No significant associations**				
**HTLV-2**	**Log*_e_* T/S ratio**	0.380	(0.176, 0.820)	0.400	(0.178, 0.897)

* Multivariable logistic regression adjusted for age, smoking (pack-years), loge (T/S ratio).
